# Optimal Stopping Rules for Preventing Overloading of Multicomponent Systems

**DOI:** 10.3390/ma16072817

**Published:** 2023-04-01

**Authors:** Andrzej Z. Grzybowski, Zbigniew Domański, Tomasz Derda

**Affiliations:** Department of Mathematics, Czestochowa University of Technology, PL-42201 Czestochowa, Poland

**Keywords:** array of pillars, failure, multicomponent system, optimal stopping rule, probability and statistics, strength

## Abstract

When random-strength components work as an interconnected parallel system, then its carrying capacity is random as well. In a case where such a multicomponent system is a subject of the stepwise-growing workload, some of its components fail and their loads are taken over by the ones that are intact. When the loading process is continued, the additional loads trigger consecutive failures that degrade the system, eventually leading to a complete failure. If the goal of the system is to carry as much load as possible, then the loading process should be continued, but no longer than until the loading capacity of the whole system is reached. On the other hand, with every additional load step, a failure of the system becomes more probable, as the carrying capacity is random and known solely through its probability distribution. In such cases, the decision on when to cease the loading process is not obvious. We introduce and analyse a minimal model of failure spreading in an array of progressively loaded pillars controlled by a decision-maker who stops the process when a required load is attained. We show how to construct an optimal stopping rule. Under some additional assumptions regarding the adopted loss function, it is argued that the optimal stopping rule is of the threshold type and it significantly depends on the shape of the load-step probability distribution.

## 1. Introduction

Knowledge of reliable and secure methods of loading that maximize the accessible carrying capacity of loaded systems are important in multiple areas of technology and logistics. Specifically, a progressive loading of a multi-component system until a satisfactory load is reached while the system’s integrity is preserved represents a complex process. This is mainly due to cascades of failed components that begin to appear when a certain load amplitude is exceeded. Each consecutive cascade is followed by a load transfer from failed components to intact ones, which in turn possibly trigger further failures. The cascades may become self-sustained, resulting either in a catastrophic wave of failures that destroy the whole system or they stop and arrest the system in a stable configuration. In various interesting problems, the system is a subject of stepwise load increment, while its integrity needs to be preserved. A decision-maker observing such a loading process sequentially is expected to maximise the load but, at the same time, to stop the process before a catastrophic cascade of failures starts to develop. They face the problem of finding the optimal stopping rule.

Arrays of pillars [1] belong to a class of electro-mechanical multicomponent systems. This class involves devices that are important and frequently encountered in such areas of nanotechnology as bio-mechanical sensing [2,3], nanoscale electronics, thermoelectrics or photovoltaics, to name but a few [4,5]. Especially prominent applications of nanopillars refer to flexible sensors capable of detecting and measuring multidirectional forces [6] or high-performance piezoelectric nanogenerators that convert external mechanical energy into electricity [7,8,9,10]. Due to size effects, sub-micron-scale pillars reveal enhanced strength when compared with their bulk counterparts [11,12]. It turns out, that after assembling a given bundle of pillars into an array with a prescribed geometry, the overall strength of such an array depends on the mutual positions of individual pillars. This means that the resulting arrays display non-negligible sample-to-sample fluctuations when subjected to an external load [13,14]. As a consequence, the carrying capacity is a random value known solely through its probability distribution. It makes the above-indicated optimal stopping problem much more complicated. This problem is especially difficult and interesting if the gain from the loaded multicomponent system monotonically increases when the total load grows, but at the same time, catastrophic failure becomes more and more probable. In such a case, the decision of when to stop the loading process is not obvious.

With this in mind, we introduce and analyse a minimal model of crushes spreading in an array of progressively loaded pillars surveyed by a decision-maker who stops the process when an optimal load is attained. An array of pillars as a model of a multi-component system under progressive load enables us to keep the model minimal but not simplistic, i.e., being capable to illustrate the main features of optimal stopping procedure yet still remaining mathematically transparent.

We restrict our study to a statistical description of the system by employing distributions of relevant microscopic quantities such as pillar-strength-thresholds (σ). Our model is formulated around a few key constituents regarding the system itself and a decision maker that observes the loading process. Namely, a rule that governs how a load released by a crushed pillar is transferred to the intact ones and a distribution of σ characterise the system, whereas a given load-step distribution together with a functional link between the applied load and resulting payoff refer to a decision-maker-action.

This work presents how to construct a reliable stopping rule in a non-deterministic case, i.e., when randomness originating from the system constituents is entangled with that related to the loading process realisation.

In the following, in Section 2, we specify pillar arrays and a compressive test enabling us to determine a correct distribution of a system’s carrying capacity. Section 3 contains a brief introduction to a so-called blackjack-type stopping problem together with necessary notions and definitions. The main results are presented in Section 4, which directly deals with the optimal stopping rules of pillar-arrays loading. Finally, we summarise our findings.

## 2. Array of Pillars under Progressive Loading

**Figure 1:**
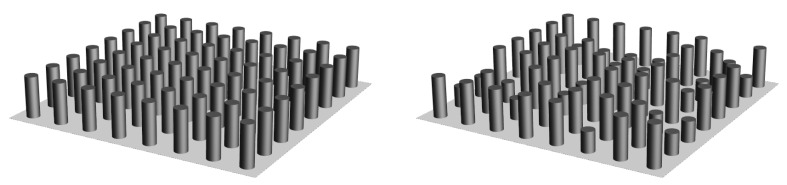
Schematic view of array of pillars: before loading (**left** panel) and under a load (**right** panel). High columns represent intact pillars and low ones refer to crushed pillars.

We consider an array of *N* pillars placed at nodes of a square substrate that transfers stresses resulting from loads felt by the pillars, see Figure 1. Since a number of pillars encountered in such nanodevices as, e.g., nanogenerators is of the order of $10^{3}$–$10^{4}$ [9,15,16], for illustration purposes we choose N=100×100.

### 2.1. Pillars

In our model, a pillar is seen as a two-state unit, either intact or crushed. The intact state refers to a fully functional pillar even though its bulk structure evolves with a growing load. When a load carried by the pillar attains a given pillar’s strength, the pillar becomes crushed irreversibly. This means that the strength threshold establishes a link between the state of the pillar and the applied load. Within such a scenario, the pillars are characterised solely by strength thresholds σ. Due to various material defects and manufacturing imperfections σ is a random variable drawn from a given probability distribution. In this paper, we adopt a widely agreed Weibull distribution. The corresponding density function reads:

(1) $$p_{\rho }\left(\sigma \right)=\rho \sigma^{\rho -1}\exp\left[-\sigma^{\rho }\right],\;\sigma >0,$$

where the shape parameter ρ reflects variations of pillar-strength-thresholds within a considered array. Besides ρ, the Weibull distribution (1) also involves the scale parameter λ, which directly tunes the distribution’s argument and the *pdf* as σ/λ and $\lambda \cdot p_{\rho }$, respectively. Since it does not change the shape of the distribution, we assume λ=1. Thus, Equation (1) holds through all our computations.

### 2.2. Load Transfer Rule

Immediately after loading, the pillars start to interact elastically through stresses, which are localised in the substrate. When a pillar *i* crushes its load, $\Delta Q_{i}$ is distributed among intact pillars in a way reflecting mechanical properties of array’s substrate and pillars’ fixations. In this context, a simple rule governing such a distribution originates from a power law relation $∼1/r^{\gamma }$ which, in turn, describes how the stress decreases at the distance *r* from a damaged location in a homogeneous material. For our purpose, we employ a so-called range variable (RV) load transfer rule [17,18] being a discrete variant of the above-mentioned power law relation. The RV rule allocates fractions of $\Delta Q_{i}$ among all intact pillars according to the following expression:

(2) $$\frac{Z_{i}}{|r_{j}-r_{i}{|}^{\gamma }}\Delta Q_{i},$$

where $|r_{j}-r_{i}|$ is the distance between crushed and intact pillars, and the normalisation factor $Z_{i}$ ensures that the load is conserved. When the adjustable power index γ varies, the RV rule smoothly interpolates between the short range (γ→∞) and long range (γ=0) interactions among pillars. The parameter γ should be tuned in accordance with the substrate rigidity.

### 2.3. Arrays’ Strengths

With a view to finding a pillar array carrying capacity ($Q_{c}$) and then, to specify distribution $g(Q_{c})$ across a given ensemble of arrays, we perform the following quasi-static compression test. A load *Q*, growing stepwise with increments sufficient to crush only the pillars closest to failure, is applied with an initial increment equal to min $\left\{\sigma \right\}$. Then, assume that under a certain load $Q^{{}^{\prime }}$, the array is in a configuration called stable, where all the pillars are intact. A consecutive incremental load raises the total load as $Q^{{}^{\prime }}\to Q^{{}^{\prime }}+\delta Q$, which induces crushes either driving the system to another stable configuration or inducing an ultimate collapse that yields $Q_{c}=Q^{{}^{\prime }}$.

In order to collect data required by a reliable estimate of $g(Q_{c})$, we generate an ensemble of $10^{4}$ arrays of pillars with chosen values of ρ and γ. Each array undergoes the compression test. Then, the resulting empirical distribution of $Q_{c}$ is verified by suitable goodness-of-fit tests, including the Cramer–von Mises and Anderson–Darling tests [19].

## 3. Blackjack-Type Optimal Stopping Problems

To solve the problem described in the Introduction, we adopt methods of the optimal stopping theory. This theory deals with the problem of choosing the best time to take a specific action. It covers a broad group of problems investigated in various branches of engineering [20,21,22,23,24]. Here, we focus on the so-called blackjack-type stopping problems, which were studied in [25,26]. This class of problems provides valuable models for tasks of preventing the overloading of multicomponent systems. To be precise in our considerations, we need to introduce some formal definitions and results.

Consider a finite sequence $q_{1},q_{2},\ldots ,q_{m}$ of independent, identically distributed random steps whose probability density f(q) is known and let $Q_{k}=q_{1}+q_{2}+\ldots +q_{k},k\leq m$ denote the total load carried by the system once *k* steps are executed. A decision maker observes sequentially the values $q_{k}$ and decides whether to stop or to continue. If they decide to stop at the moment *k*, they obtain a value $W(Q_{k})$, where a non-negative function *W* represents a defined gain. We assume that the function *W* is non-decreasing on the interval $\left(0,Q_{c}\right]$ and is non-increasing for arguments greater than $Q_{c}$. Such problems are called blackjack-type problems if the random variables $\left\{q_{i}\right\}$ are non-negative, as is the case here, and the function *W* achieves its only maximum $W(Q_{c})$, where $Q_{c}>0$ is a limit given in the problem. The decision maker has to find a stopping rule that maximises the expected gain.

In order to present solutions to blackjack-type problems, we need theoretical results from the sequential analysis theory. As they have a profoundly formal-mathematical character, we omit them. Interested readers are referred to [27,28] for the details, including the formal definition of a stopping rule *Q*. Roughly speaking, the definition assures that at each step the decision maker knows whether to stop or not solely on the basis of the previous observations. Another important notion that we need here is the value *V* of the stopping problem. It is simply the greatest expected gain that can be achieved in a given stopping problem by the usage of the optimal stopping rule $Q^{*}$.

The following Proposition, which we have proved in [25], states that in many interesting cases, the optimal stopping rule is of the threshold type, i.e., it is a constant that does not depend on the number of already observed variables, and regardless of whether the assumed number of possible steps is bounded or not. We present here its version that addresses directly the problem of stopping the loading process.

**Proposition 1.**  *Let $q_{1},q_{2},\ldots ,q_{m}$ be independent, identically distributed random load steps and let*

(3) $$V_{1}\left(Q\right)=\int_{0}^{\infty }W\left(Q+q\right)f\left(q\right)dq.$$

If there exists a real number $Q^{*}$, $0<Q^{*}<Q_{c}$, such that

(4) $$W\left(Q\right)<V_{1}\left(Q\right)\;\mathrm{for}\;0\leq Q<Q^{*}\;\mathrm{and}\;W\left(Q\right)\geq V_{1}\left(Q\right)\;\mathrm{for}\;Q\geq Q^{*}$$

*then the optimal stopping rule for the problem is given by the following formulae:*

(5) $$Q^{*}=\min\left\{1\leq k\leq m:Q_{k}\geq Q^{*}\right\}.$$

The above defined function $V_{1}$ can be interpreted as the expected gain in a case where the state of the loading process is *Q* and we have only one loading step *q* ahead. We emphasize once again that the essential feature of the above stopping rule is its threshold character, and this threshold $Q^{*}$ does not depend on the presumed maximal number of steps.

The crucial aspect of the blackjack-type problems considered above was the deterministic character of the problem-limit $Q_{c}$, the limit is assumed to be known. In our main problem, however, related to the loading process of the arrays of pillars, this limit is unknown. More precisely, all we know is the probability distribution of the load-capacity-limit, which can be approximated with the help of the Monte Carlo experiments [13]. Such a change in assumptions needs the extension/modification of the original blackjack-type problem.

So, now we turn our attention to a problem where the given constant limit number is replaced by a random variable with a known probability distribution. In such a case, while “stepping forward”, the decision-maker does not know how far the actual border that should not be crossed is. They only know the probabilities about the limit specific ranges. This is why we need to re-define the gain function. We want to maintain, however, the same spirit of the problem. The fundamental feature of the blackjack-problem can be more generally yet briefly stated as follows: with every single step, the possible gain becomes larger, but at the same time, possible punishment becomes more probable. Thus, we propose to adopt the following model for this situation.

Let $Q_{c}$ be a random variable with known probability density function $g(Q_{c})$. Let Re:R→R be a non-decreasing function that represents a *reward* for the decision-maker, while Pu:R→R is a non-increasing function that represents a *punishment*. For each load *Q*, Re(Q)>Pu(Q). The decision-maker receives either Re(Q) or Pu(Q), dependently on whether or not *Q* is greater than $Q_{c}$. Because $Q_{c}$ is a random variable, the decision-maker receives Re(Q) or Pu(Q) with probabilities that result from the probability distribution of $Q_{c}$. Thus, the overall payoff *Z* is the following expected value of such a “lottery”:

(6) $$Z\left(Q\right)=Re\left(Q\right)\int_{Q}^{\infty }g\left(x\right)dx+Pu\left(Q\right)\int_{-\infty }^{Q}g\left(x\right)dx$$

The decision-maker’s task is to find an optimal stopping rule in such a situation. The proposed model will be called a blackjack-type stopping problem with a random limit. In order to make it easier to distinguish between the two situations, the function *W* that represents a payment for the decision-maker in the deterministic case, as so far, will be called *gain* function, whilst the function *Z* defining the payment in the random case will be called the *payoff* function. It turns out that in many interesting cases connected with the process of loading the arrays of pillars, the optimal stopping problem with a random limit is equivalent to the original deterministic blackjack-type stopping problem.

## 4. Optimal Stopping Rules for a Pillar-Array Loading

In this section, we present a specific example illustrating the possible application of the above introduced theoretical model and results. We consider a problem of loading the arrays determined by the following structural parameters: N=100×100 pillars, whose random strength-thresholds σ are distributed according to Equation (1) with the shape parameter ρ=2. In our simulations, the RV load transfer rule, given by Equation (2), operates in a regime characterized by γ=4 which, in turn, corresponds to a short-range-like pillar-to-pillar interactions [14].

### 4.1. Load Limit and Payoff Function

**Figure 2:**
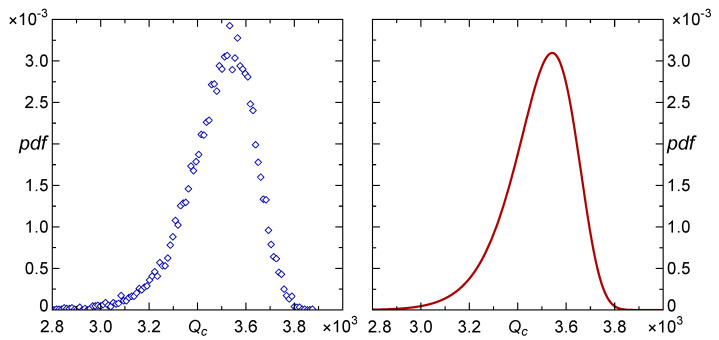
Empirical distribution of $Q_{c}$ obtained from $10^{4}$ arrays of 100×100 pillars (**left** panel) and the resulting probability density of the Weibull distribution, see Equation (7), with parameters estimated from the simulations (**right** panel).

For such pillar arrays, the distribution of their carrying capacity ($Q_{c}$) was determined with the help of simulation experiments presented in Section 2.3, as well as described in greater detail in [13,14]. The resulting distribution is displayed in Figure 2, see the left panel. It appears that the empirical distribution of $Q_{c}$ can be very accurately approximated by the three-parameter Weibull density

(7) $$g\left(Q_{c}\right)=\left\{\begin{array}{cc} \frac{\alpha }{\beta }{\left(\frac{Q_{c}-Q_{0}}{\beta }\right)}^{\alpha -1}\exp{\left[-\frac{Q_{c}-Q_{0}}{\beta }\right]}^{\alpha }, & \;\mathrm{for}\;Q_{c}\geq Q_{0},\\ 0, & \;\mathrm{elsewhere} \end{array}\right.$$

where the shape, scale and location parameters are equal, respectively: α=14.23,β=1694.58 and $Q_{0}=1856.22$. In this case, the mean value ${\bar{Q}}_{c}=Q_{0}+\beta \Gamma \left(\frac{1}{\alpha }+1\right)=3489.84$. The distribution $g(Q_{c})$ is presented in the right panel of Figure 2.

The above density g(Q) enables us to compute the corresponding survival function $S\left(Q\right)=1-\int_{0}^{Q}g\left(x\right)dx$, which represents the probability that a given array safely supports an applied load *Q* [14]. Namely:

(8) $$S\left(Q\right)=\left\{\begin{array}{cc} 1, & \;\mathrm{for}\;0\leq Q<Q_{0},\\ \exp{\left[-\frac{Q-Q_{0}}{\beta }\right]}^{\alpha }, & \;\mathrm{for}\;Q\geq Q_{0}. \end{array}\right.$$

**Figure 3:**
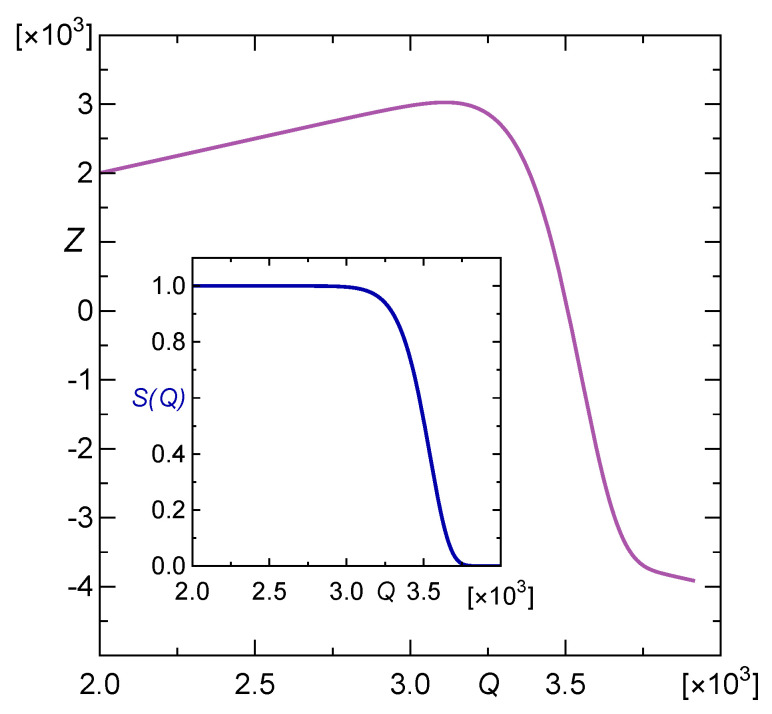
Payoff function *Z* given by Equation (10). The inset shows survival function *S* given by Equation (8).

Now, in order to determine the optimal stopping rule, we need to know the payoff function defined by Equation (6). For our illustrative purpose, we assume that the reward part is proportional to the total load put on the array, while the punishment part (activated in case of overloading) is the opposite number. Without loss of generality, the proportion coefficient can equal 1. Hence, in our example,

(9) Re(Q)=Q,andPu(Q)=−Q.

and resulting payoff function

(10) $$Z\left(Q\right)=Q\left\{2\cdot \exp\left[-{\left(\frac{Q-Q_{0}}{\beta }\right)}^{\alpha }\right]-1\right\}$$

is presented in Figure 3. We recall that the general formula defining *Z* is given by Equation (6).

Its global maximum 3025.1 is attained at $Q_{\max}=3111.4$. The function is increasing on the interval $\left(0,Q_{\max}\right]$ and is decreasing for arguments greater than $Q_{\max}$. So, we deal with the blackjack-type optimal stopping problem. In our problem, an array is loaded sequentially, by applying random load steps, one by one, until the rule tells us to stop, i.e., until we cross the threshold $Q^{*}$. The last thing needed to find the value of the threshold $Q^{*}$ is the probability distribution f(q) of the load-steps $q_{1},q_{2},\ldots$, that will be applied on the pillar array during the loading process. In accordance with Equation (4), $Z<V_{1}$ for $Q<Q^{*}<Q_{\max}$ and inversely, $Z>V_{1}$ for $Q^{*}<Q<Q_{\max}$. Therefore, $Q^{*}$ corresponds to the unique solution of the following integral equation:

(11) $$Z\left(Q\right)=\int_{0}^{\infty }Z\left(Q+q\right)f\left(q\right)dq.$$

**Figure 4:**
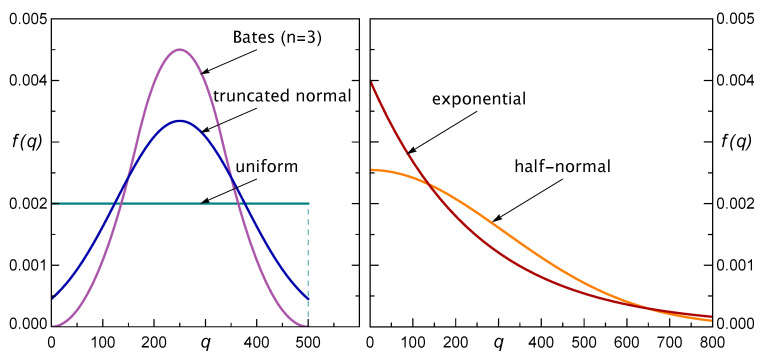
Sketch of considered load steps distributions f(q), as drawn in the case $\bar{q}=250$. **Left** panel: PDFs with the finite support $\left[0,\bar{q}\right]$. **Right** panel: PDFs with the semi-infinite support $\left[0,\infty \right]$.

We will distinguish five illustrative distributions f(q): uniform, Bates distribution for n=3, truncated normal, half-normal, and exponential ones. Their parameters were chosen so that the mean value $\bar{q}$ of the single step was the same. In this regard, we present the results for five cases where $\bar{q}\in \left\{150,175,200,225,250\right\}$. Figure 4 shows these distributions sketched for $\bar{q}=250$.

### 4.2. Uniform Distribution of Load Steps

**Figure 5:**
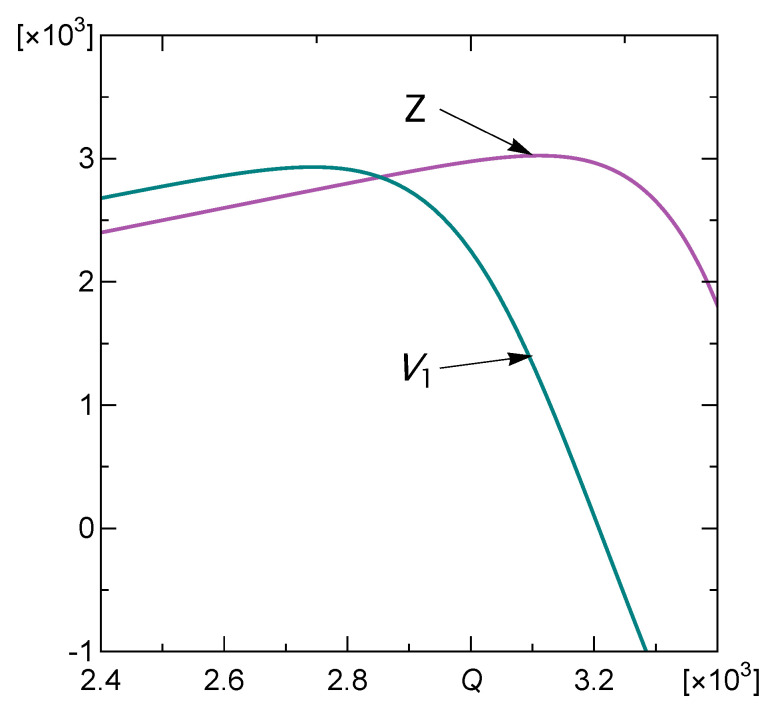
The function $V_{1}$ and the payoff function *Z* in the case of uniformly distributed loading steps with $\bar{q}=250$.

Let us assume that the load steps are distributed according to uniform distribution defined on the interval $\left[0,500\right]$. In this case, the function $V_{1}$ (from Proposition) cannot be obtained explicitly, but its values can be determined numerically, and it can be verified that the condition Equation (4) holds. The function $V_{1}$ along with the payoff *Z* are presented in Figure 5.

It results from the Proposition that to find the corresponding threshold $Q^{*}$ determining the optimal stopping rule, see Equation (5), we need to solve Equation (11). Although $V_{1}$ is given only in the integral form, the solution can be easily found numerically. In our example, it equals $Q^{*}=2872.45$. So, our stopping rule tells us to continue the process of loading the array of pillars until the total load is greater than (or equal to) $Q^{*}=2872.45$ for the first time.

### 4.3. Solution of Optimal Stopping Problem

Typically, the solution of the optimal stopping problem consists of the optimal stopping rule, which we have already found, and the value of the problem. Again, in the considered case, it can only be performed numerically. Our proposal is to use a proper Monte Carlo procedure that was already applied in similar tasks, see [26]. A procedure that can be used for our problem may follow the steps:

1. Set Load=0
2. Set ActualLimit = RandomNumber[LimitDistribution]
3. While (Load<Q*)AND(Load<ActualLimit) set
   
Load=Load+RandomNumber[StepDistribution]
4. If Load < ActualLimit set Payoff = Load else set Payoff = -Load
5. Return Payoff

After simulating $10^{5}$ runs of the above procedure, we found the approximate value of the problem, which equals 2944.18.

Analogous results can be obtained for the optimal stopping problems with step distributions presented in Figure 4. More precisely, apart from the uniform one, we also considered the following step distributions: Bates distribution with n=3 and the support $\left[0,b\right]$, normal distribution N(b/2,b/4) truncated to the interval $\left[0,b\right]$ (in both cases $b\in \left\{300,350,400,450,500\right\}$), and half-normal and exponential distributions, both with a mean $\bar{q}\in \left\{150,175,200,225,250\right\}$. Such a choice of the parameters of the distributions assures that the mean value of the single step was the same for all considered distribution types. The results presented in Table 1 were received in precisely the same way as described in Section 4.2 for the uniformly distributed steps.

Before the analysis of the presented results, let us note that, among the considered step distributions, we have three distributions that are defined on finite support, and two with semi-infinite support. The first three are uniform, Bates, and truncated normal, while the latter ones are half-normal and exponential. The density functions of these distributions are shown in Figure 4.

Now, based on Table 1, one can make several interesting observations regarding our stopping problem.

The first observation is rather natural and expected; our results confirm that for any fixed type of distribution, the longer the step (as represented by its expected value $\bar{q}$), the lower the value of the corresponding thresholds for stopping. It is natural because with long random steps, being close to the verge of loading capacity, one can much easier cross the unknown limit, while with short steps one could “carefully” try to move closer to the limit. Such an intuitive interpretation is confirmed formally by our theoretical results. What is perhaps less obvious, however, is that such a relationship between the step length and the threshold value can be strongly violated if, along with the length of the steps, also the type of its distribution changes. For instance, the threshold in the case of steps having Bates distribution with $\bar{q}=250$ is greater than the one for steps with uniform distribution with a mean of $\bar{q}=225$. It is so even in spite of that in the case of uniform distribution, the steps have a smaller maximum length. The same is true for other pairs of successive step lengths presented in Table 1. The possible reason is the shape of the distributions, see Figure 4. We see that, in the case of uniform distribution, the greatest values, which are close to the right endpoint of the interval, are much more probable than the same values in the case of Bates distribution. This indicates that the shape of the step distribution has a significant impact on this relation.

Another observation concerns the impact of the variability of the step lengths (represented by their standard deviations) on the optimal threshold value. One could expect that for a fixed average-step-length the increment of the dispersion should result in a smaller value of the optimal threshold. Intuitively, it should be so as a result of greater uncertainty connected with the actual step lengths. Such uncertain steps may be riskier in the neighbourhood of the loading capacity limit. In our problems, this relation is true for all examined types of step distribution. It indicates that: (i) the value of the optimal stopping threshold is sensitive to the uncertainty resulting from the dispersion of the step lengths, and (ii) this value tends to become smaller when the uncertainty increases.

Our next observations refer to the value of the problem. As we know, this is a very important characteristic for a decision-maker, because it shows the maximal expected payoff that could be gained in a given specific problem of an array-of-pillars loading. The results in Table 1 show that for each type of step distribution, the value of the problem increases when the average length of steps decreases. On the other hand, based on our results, similar monotonic relationship can be also observed between the values of the problem and the standard deviation of the step lengths. Both observations are easy to explain. However, the joint effect of changes in step lengths and their variability is not clear. It certainly depends on the degree of relative changes in those parameters and on the type of step distribution. For instance, when comparing the Bates distribution with $\bar{q}=250$ with half-normal distribution with $\bar{q}=150$, we see that the value of the problem is greater for the first one (although the step is much greater on average). In contrast, the value of the problem for the truncated normal distribution with $\bar{q}=250$ is less than the one for uniform distribution with $\bar{q}=200$, in spite of a greater standard deviation of the latter. Again, we see that apart from those two basic statistical measures, the type of the specific step distribution plays an important role. All our observations confirm that in the considered problem of preventing the overloading of the arrays, it is essential to acquire more detailed knowledge about the specific type and shape of the step distribution. Such knowledge is necessary to develop a proper stopping rule.

## 5. Conclusions

We have investigated how to establish a stopping rule that allows one to approach a high level of applied load and, at the same time, to protect the loaded system from a catastrophic failure. The results point out some important facts about the problem.

First, let us note that it would be unwise to adopt a naive stopping policy: continue the loading process until one reaches the mean carrying capacity [29]. In all cases presented in Table 1, the optimal stopping threshold is much less than that value. What is also interesting is that neither the mean nor the standard deviation of the random step provides enough information for the proper decision about when to stop the loading process. We can see that the values of the problem, i.e., the expected maximal payoff, do not depend on those parameters in an obvious way. Namely, the same mean value of the step results in significantly different optimal stopping thresholds as well as different expected maximal payoffs depending on the shape of considered distributions. On the other hand, although the relationship between these values and the standard deviations of the steps seems to be monotonic, it is still not enough to draw a conclusion about the optimal threshold value in a specific problem. This indicates that what matters here is the type of step distribution. It is often encountered in engineering praxis to look merely at the two principal parameters of the random distributions: the mean and the standard deviation. As we see it would be not enough in the above-considered decision-making tasks.

Our study clearly shows that for a given multicomponent system, the optimal threshold and the value of the problem strongly depend on the shape of the loading step distribution. This remark is very important, since it indicates the bounds of the direct applicability of the results.

The limitations of the potential applications of the presented above optimal stopping rules result from the latter remark, i.e., the proposed method is reasonably applicable only if one can acquire detailed knowledge of the probability density function of the loading step distribution [29,30]. Obviously, such a requirement also concerns the distribution of the limit of the loading capacity of the system. Thus, in engineering praxis, when dealing with similar problems, one should conduct an appropriate statistical study to determine the shapes of those distributions as precisely as possible. It is quite a restrictive requisite from the perspectives of many potential applications. However, if it can be satisfied, then the effort is compensated by the procedure that optimally prevents system overloading.

## Figures and Tables

**Table 1:** Data corresponding to optimal stopping with chosen loading step distributions.

Step Distribution	Mean Load Step $\bar{q}$	Step Standard Deviation	Optimal Threshold $Q^{*}$	Value of the Problem *V*
Bates	250	83.33	2927.98	2975.70
truncated normal	250	109.95	2905.02	2963.97
uniform	250	144.34	2872.45	2944.18
half-normal	250	188.88	2760.99	2827.05
exponential	250	250.00	2600.77	2600.55
Bates	225	75.00	2950.35	2984.43
truncated normal	225	98.95	2930.82	2973.76
uniform	225	129.90	2902.56	2960.94
half-normal	225	169.99	2805.83	2861.57
exponential	225	225.00	2657.74	2658.53
Bates	200	66.67	2971.88	2992.69
truncated normal	200	87.96	2955.56	2983.30
uniform	200	115.47	2931.45	2973.25
half-normal	200	151.10	2849.89	2895.03
exponential	200	200.00	2715.57	2714.77
Bates	175	58.33	2992.52	3001.67
truncated normal	175	76.97	2979.17	2994.49
uniform	175	101.04	2959.05	2985.46
half-normal	175	132.21	2892.68	2926.08
exponential	175	175.00	2773.93	2771.59
Bates	150	50.00	3012.26	3007.89
truncated normal	150	65.97	3001.62	2999.43
uniform	150	86.60	2985.26	2997.42
half-normal	150	113.33	2933.63	2952.24
exponential	150	150.00	2832.40	2831.59

## Data Availability

Not applicable.

## References

[1] Chekurov N., Grigoras K., Peltonen A., Franssila S., Tittonen I. (2009). The fabrication of silicon nanostructures by local gallium implantation and cryogenic deep reactive ion etching. Nanotechnology.

[2] Park J.E., Won S., Cho W., Kim J.G., Jhang S., Lee J.G., Wie J.J. (2021). Fabrication and applications of stimuli-responsive micro/nanopillar arrays. J. Polym. Sci..

[3] Harding F.J., Surdo S., Delalat B., Cozzi C., Elnathan R., Gronthos S., Voelcker N.H., Barillaro G. (2016). Ordered Silicon Pillar Arrays Prepared by Electrochemical Micromachining: Substrates for High-Efficiency Cell Transfection. ACS Appl. Mater. Interfaces.

[4] Schoen I., Hu W., Klotzsch E., Vogel V. (2010). Probing Cellular Traction Forces by Micropillar Arrays: Contribution of Substrate Warping to Pillar Deflection. Nano Lett..

[5] Qiu X., Lo J.C.C., Lee S.W.R., Liou Y.-H., Chiu P. Evaluation and Benchmarking of Cu Pillar Micro-bumps with Printed Polymer Core. Proceedings of the 2019 International Conference on Electronics Packaging (ICEP).

[6] Chen X., Shao J., Tian H., Li X., Tian Y., Wang C. (2018). Flexible three-axial tactile sensors with microstructure-enhanced piezoelectric effect and specially-arranged piezoelectric arrays. Smart Mater. Struct..

[7] Chen X., Li X., Shao J., An N., Tian H., Wang C., Han T., Wang L., Lu B. (2017). High-Performance Piezoelectric Nanogenerators with Imprinted P(VDF-TrFE)/BaTiO3 Nanocomposite Micropillars for Self-Powered Flexible Sensors. Small.

[8] Choi Y.-Y., Yun T.G., Qaiser N., Paik H., Roh H.S., Hong J., Hong S., Han S.M., No K. (2015). Vertically aligned P(VDF-TrFE) core-shell structures on flexible pillar arrays. Sci. Rep..

[9] Mervat I., Jinxing J., Zhen. W., Xuhui S. (2021). Surface Engineering for Enhanced Triboelectric Nanogenerator. Nanoenergy Adv..

[10] Rakotondrabe M., Yang R., Wang L.Z. (2022). Editorial for the Special Issue on Piezoelectric Nanogenerators for Micro-Energy and Self-Powered Sensors. Micromachines.

[11] Greer J.R., Jang D., Kim J.-Y., Burek J. (2009). Emergence of New Mechanical Functionality in Materials via Size Reduction. Adv. Funct. Mater..

[12] Jang D., Greer J.R. (2010). Transition from a strong-yet-brittle to a stronger-and-ductile state by size reduction of metallic glasses. Nat. Mater..

[13] Derda T., Domanski Z. (2020). Enhanced strength of cyclically preloaded arrays of pillars. Acta Mech..

[14] Derda T., Domanski Z. (2021). Survivability of Suddenly Loaded Arrays of Micropillars. Materials.

[15] Zhu Y., Yang B., Liu J., Wnag X., Wang L., Yang C. (2016). A flexible and biocompatible triboelectric nanogenerator with tunable internal resistance for powering wearable devices. Sci. Rep..

[16] Shin S.-H., Choi S.-Y., Lee M.H., Nah J. (2017). High-Performance Piezoelectric Nanogenerators via Imprinted Sol–Gel BaTiO_3_ Nanopillar Array. ACS Appl. Mater. Interfaces.

[17] Hidalgo R.C., Moreno J., Kun F., Herrmann H.J. (2002). Fracture model with variable range of interaction. Phys. Rev. E.

[18] Roy S., Biswas S., Ray P. (2017). Modes of failure in disordered solids. Phys. Rev. E.

[19] Arnold T., Emerson J. (2011). Nonparametric goodness-of-fit tests for discrete null distributions. R J..

[20] Wu Y. (2023). Optimal Stopping and Loading Rules Considering Multiple Attempts and Task Success Criteria. Mathematics.

[21] Qiu Q., Cui L. (2018). Reliability evaluation based on a dependent two-stage failure process with competing failures. Appl. Math. Model..

[22] Oosterom C.D., Elwany A.H., Çelebi D., Houtum G.J. (2014). Optimal policies for a delay time model with postponed replacement. Eur. J. Oper. Res..

[23] Liu X., Wang W., Peng R., Zhao F. (2015). A delay-time-based inspection model for parallel systems. J. Risk Reliab..

[24] Sun Y.T., Liu C., Zhang Q., Qin X.R. (2017). Multiple Failure Modes Reliability Modeling and Analysis in Crack Growth Life Based on JC Method. Math. Probl. Eng..

[25] Grzybowski A.Z., Korsunsky A. (2010). Optimal Stopping Rules For Some Blackjack Type Problem. Current Themes in Engineering Science.

[26] Grzybowski A.Z. (2011). Monte Carlo Analysis of Risk Measures for Blackjack Type Optimal Stopping Problems. Eng. Lett..

[27] Chow Y.S., Robbins H.E., Siegmund D. (1971). Great Expectations: The Theory of Optimal Stopping.

[28] Shiryaev A.N. (2008). Optimal Stopping Rules.

[29] Cha J.M., Mi J. (2007). Study of a stochastic failure model in a random environment. J. Appl. Probab..

[30] Yang L., Peng R., Zhao Y. (2018). Hybrid preventive maintenance of competing failures under random environment. Reliab. Eng. Sys. Saf..

